# Regulation of meiotic gene expression in plants

**DOI:** 10.3389/fpls.2014.00413

**Published:** 2014-08-25

**Authors:** Adele Zhou, Wojciech P. Pawlowski

**Affiliations:** School of Integrative Plant Sciences, Cornell University, Ithaca, NYUSA

**Keywords:** meiosis, transcriptome, gene expression, gene regulation, chromatin, non-coding RNAs

## Abstract

With the recent advances in genomics and sequencing technologies, databases of transcriptomes representing many cellular processes have been assembled. Meiotic transcriptomes in plants have been studied in *Arabidopsis thaliana*, rice (*Oryza sativa*), wheat (*Triticum aestivum*), petunia (*Petunia hybrida*), sunflower (*Helianthus annuus*), and maize (*Zea mays*). Studies in all organisms, but particularly in plants, indicate that a very large number of genes are expressed during meiosis, though relatively few of them seem to be required for the completion of meiosis. In this review, we focus on gene expression at the RNA level and analyze the meiotic transcriptome datasets and explore expression patterns of known meiotic genes to elucidate how gene expression could be regulated during meiosis. We also discuss mechanisms, such as chromatin organization and non-coding RNAs that might be involved in the regulation of meiotic transcription patterns.

## INTRODUCTION

In contrast to the predetermined germline of animals, plants spend most of their life cycle maintaining a limited population of undifferentiated stem cells that are activated to become the germline only for a brief time in the plant’s life (Berger and Twell, 2011). This process involves a switch in cell identity, which necessitates a distinct change in gene expression patterns (Berger and Twell, 2011). Establishing and maintaining the fate of meiotic cells must then require intricate regulation of gene expression at the transcriptional level. Progressing through meiosis, a hallmark of sexual reproduction (see Ma, 2006; Jones and Franklin, 2008), also necessitates a distinct pattern of gene expression. As meiosis encompasses unique processes that do not occur in vegetative cells, it requires expression of genes different from those needed in non-meiotic tissues.

A challenge for the regulation of meiotic gene expression at the RNA level, which is a subject of this review, is that it must be initiated and maintained when chromosomes are already condensed and access to chromatin becomes limited. Numerous other demands are also placed on chromatin at that time. Chromatin structure is critical for homologous chromosome pairing and meiotic recombination (Dawe et al., 1994; Golubovskaya et al., 2006). For example, the SPO11 complex, which initiates meiotic recombination, acts on chromatin loops during meiotic prophase I (Blat et al., 2002). At that time, chromosomes have already started condensing, so mechanisms must exist to allow selective access to specific chromosome sites that undergo recombination. However, pairing and recombination cannot interfere with the ability of essential genes to be expressed. Thus, a regulatory network operating during meiosis has to coordinate the occurrence of chromatin states required for meiotic events with those needed for gene expression.

Surprisingly, there is limited understanding of how the complexity of gene expression in meiosis is regulated. Particularly little information is available from plants. In this review, we will describe data on meiotic transcriptomes from studies conducted in several plant species, as well as data on expression patterns of known meiotic genes. Based on these studies, we will draw hypotheses and speculations about mechanisms that are likely involved in regulating meiotic gene expression in plants.

## THE MEIOTIC TRANSCRIPTOME

Global transcriptome studies, which present information on gene expression patterns, provide an efficient platform for elucidating the mechanisms underlying meiotic gene regulation. Within the past decade, a number of transcriptome analyses of meiotic tissues have been conducted in plants, and provide valuable insight into how gene expression in meiosis is controlled. Meiotic transcriptomes have been studied in a variety of species, including *Arabidopsis*, rice, wheat, petunia, sunflower, and maize (Cnudde et al., 2006; Crismani et al., 2006; Chen et al., 2010; Deveshwar et al., 2011; Yang et al., 2011; Dukowic-Schulze et al., 2014a; Flórez-Zapata et al., 2014). Most of these studies have primarily focused on male meiosis, as male meiocytes in plants are more easily accessible and greater in number than female meiocytes. Additionally, due to the difficulty of extracting larger quantities of meiocytes, most transcriptome studies have used whole anthers (Cnudde et al., 2006; Crismani et al., 2006; Ma et al., 2008; Deveshwar et al., 2011). However, recently a number of studies using isolated meiocytes have been conducted as well (Chen et al., 2010; Yang et al., 2011; Dukowic-Schulze et al., 2014a,b; Flórez-Zapata et al., 2014). Though technically more challenging, utilizing isolated meiocytes eliminates confounding patterns of transcription from somatic cells, and provides a more comprehensive view of cellular activities at the time of meiosis. Analysis of isolated meiocytes also offers insights into single-tissue-type expression patterns, which are still relatively rare in plants.

Older meiotic transcriptome studies used expression microarrays, which limits results to previously annotated genes. Newer studies utilize the less biased RNA-seq approach, which, in addition to the identification of expressed genes, allows for gene discovery, as well as identification of novel splicing variants. RNA-seq is also more sensitive than microarrays in detecting low levels of gene expression. RNA-seq studies using isolated meiocytes in *Arabidopsis* and maize indicate that a surprisingly large number of genes are expressed during meiosis. In *Arabidopsis* meiocytes, approximately 20,000 genes are transcriptionally active (Chen et al., 2010; Yang et al., 2011). These genes constitute roughly 60% of the annotated genes in this species. In maize, about 50% of the 32,500 annotated genes are transcriptionally active during meiosis (Dukowic-Schulze et al., 2014b) The number of genes expressed in meiosis is similar to that in seedlings, which unlike meiocytes, contain multiple tissue types. It is, however, substantially higher than the 25% of annotated genes that are expressed during pollen development in both maize and *Arabidopsis* (Ma et al., 2008). About half of the meiotically expressed genes are expressed at significantly higher levels in meiocytes than in seedlings, in both maize and *Arabidopsis* (Chen et al., 2010; Dukowic-Schulze et al., 2014c).

Due to the nature of the two techniques, it is difficult to directly compare the numbers of expressed genes in microarray and RNA-seq studies. However, microarray studies also indicate a high number of meiotically expressed genes. In rice, microarray studies identified 2155 genes expressed at higher levels in meiotic anthers compared to seedlings. Many of these genes have not been previously linked to meiosis (Wang et al., 2005). A microarray study in petunia also found several novel meiotic genes (Cnudde et al., 2006).

Meiotic transcriptome data indicate that in addition to nuclear protein-coding genes, transposable elements and mitochondria-encoded genes are expressed during meiosis (Chen et al., 2010; Yang et al., 2011; Dukowic-Schulze et al., 2014a). Roughly 1,000 transposable elements, 32.5% of all transposable elements annotated genome-wide, are expressed in *Arabidopsis* meiocytes (Chen et al., 2010). Transposable elements belonging to the Copia, Gypsy, and SINE families exhibit the highest meiotic activity (Yang et al., 2011). Elevated transposon expression during meiosis is surprising, as intuitively, it could pose harm to the genome at a stage when preserving structural integrity of genetic material is critical. The expression of transposons may be an unintended side effect of the reorganization of chromatin structure that takes place during early meiotic prophase and is thought to facilitate key meiotic processes (Dawe et al., 1994). It is possible that transposon activity does not need to be tightly controlled during meiosis. Its presence may not be as harmful as in somatic cells, as DNA repair machinery is already upregulated at that time because many DNA repair genes also act in meiotic recombination.

Meiotic transcriptomes have been studied more extensively outside of plants, in species such as budding yeast, fission yeast, mouse, and humans (Chu et al., 1998; Primig et al., 2000; Schlecht et al., 2004; Pang et al., 2006; Chalmel et al., 2007; Wilhelm et al., 2008; Brar et al., 2012). Microarray studies in budding yeast (*Saccharomyces cerevisiae*) have identified over 1000 protein-coding genes, ∼16% of all yeast genes, that are expressed at significantly higher levels during sporulation, which is the reproductive process encompassing meiosis (Chu et al., 1998; Primig et al., 2000; Cnudde et al., 2006). In fission yeast (*Schizosaccharomyces pombe*), over 2000 genes, ∼40% of the entire gene complement of this species, were up-regulated during sporulation. Interestingly, very few of these genes are the same as the genes expressed during sporulation in *S*. *cerevisiae* (Mata et al., 2002). This observation suggests that in addition to meiotic processes conserved across eukaryotes, sporulation, including meiosis, involves processes that are more evolutionarily divergent and require expression of different genes in different species.

Extensive studies have also been performed in mouse spermatogenesis, during which around 6000 meiotically expressed genes, ∼26% of all mouse genes, were identified (Margolin et al., 2014). Mouse RNA-seq studies using whole testis showed that, in addition to genes, a substantial number of regions annotated as intergenic, undergo transcription in meiosis (Soumillon et al., 2013). Furthermore, the number of intergenic transcripts in testis was higher than in any other tissue. These transcripts could represent novel unannotated genes or novel non-coding RNAs.

The genes expressed in meiosis exhibit diverse functions and are not easy to classify in just a few distinct functional groups. However, in many studies, it has been possible to identify clusters of co-expressed genes. These clusters reveal temporal expression patterns, characterized by expression waves that are likely associated with specific stages and events of meiosis (Chu et al., 1998; Mata et al., 2002; Yu et al., 2003; Cnudde et al., 2006; Crismani et al., 2006; Ma et al., 2008; Deveshwar et al., 2011; Margolin et al., 2014). Early wave genes generally act in S phase, meiotic recombination, and homologous chromosome pairing. The mid and late waves are enriched in genes acting in the transition from prophase I to metaphase and in meiotic divisions. Temporal expression patterns could be a useful source of information on the roles of meiotic genes, by indicating in which meiotic process a specific gene is likely to function. However, expression datasets with a sub-stage level of temporal resolution do not yet exist for any plant species.

Transcriptome data are also a good starting point for elucidating regulatory pathways controlling expression of meiotic genes. Upregulation of known transcription factors and other expression regulators, which were found in *Arabidopsis* and maize transcriptome studies (Yang et al., 2011; Dukowic-Schulze et al., 2014a), could be used to pinpoint specific regulatory pathways that act in meiosis. For example, zinc finger-like proteins, known to play important roles in floral tissue development, have been found to exhibit higher expression levels in maize anthers (Ma et al., 2008). This observation suggests that they could also serve as regulators of meiotic gene expression. Mining transcription factors could provide insight into whether the massive extent of gene expression during meiosis is highly regulated, or is an unintended side effect of meiosis-specific chromatin reorganization (see “Regulation of the Meiotic Transcriptome” and “Chromatin Organization”).

## EXPRESSION PATTERNS OF KNOWN MEIOTIC GENES

A helpful approach in elucidating the regulatory networks that control meiotic gene expression is analyzing expression patterns of genes with known meiotic functions. While some meiotic genes function only in meiosis, others also function in other processes and, consequently, are expressed in other tissues and developmental stages. For example, several meiotic recombination genes also act in somatic DNA repair and are transcribed in fast-dividing meristematic tissues where spontaneous DNA damage is likely (Doutriaux et al., 1998; Grelon et al., 2001). Therefore, exclusive expression during meiosis may not be the most appropriate criterion for classifying meiotic genes. Examining expression patterns of known meiotic genes may be of help in identifying better criteria.

A potential obstacle to dissecting expression patterns of known meiotic genes is that the number of genes whose specific functions in meiosis are well understood is still relatively small. To date, only about 90 genes are documented to act in *Arabidopsis* meiosis. About 50 of them are known to be essential for meiosis (Ma, 2006; Mercier and Grelon, 2008; Yang et al., 2011; Crismani and Mercier, 2013). Of the genes with essential meiotic functions, all are highly expressed in meiocytes, but most are also expressed in other tissues (Chen et al., 2010; Yang et al., 2011). For example, *ASY1* in *Arabidopsis*, which encodes a protein essential for homologous chromosome synapsis, is expressed in both reproductive and non-reproductive tissues, though the protein is only detected in meiocytes (Caryl et al., 2000; Armstrong et al., 2002). Other genes in this category are *AHP2*, which encodes the *Arabidopsis* homolog of the yeast Hop2 protein involved in meiotic recombination and homologous chromosome pairing (Schommer et al., 2003), the *Arabidopsis* homolog of *SPO11*, which codes for a DNA topoisomerase-like protein initiating DSB formation (Grelon et al., 2001), and maize *PHS1*, which encodes a protein required for homologous chromosome pairing and recombination (Pawlowski et al., 2004). On the other hand, expression of *DMC1*, a gene encoding a recombination protein that acts only in meiosis, is restricted to meiotic cells in anthers and carpels (Klimyuk and Jones, 1997; Doutriaux et al., 1998; Couteau et al., 1999; Li et al., 2012). A third category of meiotic genes are those that are not only expressed in somatic tissues, but also in fact function outside of meiosis. Examples of such genes are those encoding components of the meiotic recombination pathway that also act in somatic DNA repair. For instance, *RAD51* in *Arabidopsis* and maize, which encodes a protein facilitating DNA strand-exchange in meiotic recombination and somatic homologous recombination, is expressed predominantly in meiotic cells as well as developing embryos and seedlings (Doutriaux et al., 1998; Franklin et al., 1999).

## REGULATION OF THE MEIOTIC TRANSCRIPTOME

Plants exhibit a greater fraction of genes that are expressed during meiosis than other species, including yeast and mammals. Consequently, it is an intriguing question whether and how meiotic gene expression is regulated in plants. There are multiple hypotheses regarding the transcriptome abundance in meiosis (Kleene, 2001). One suggests that all meiotically expressed genes indeed function in meiosis. This would indicate that the number of proteins with meiotic function is much higher than presently known, and that our current understanding of meiotic processes is extremely limited. A potential biological implication of a very large number of genes involved in meiosis would be a need for complex regulation to coordinate the timing of expression and function of all the meiotic genes and proteins. However, it seems unlikely that as many as 20,000 genes are needed for meiosis in *Arabidopsis*. On the other hand, there is evidence that many more genes are involved in meiosis than the relatively few identified so far. For example, meiotic expression of genes of mitochondrial origin, which were found to be up-regulated in meiocytes of *Arabidopsis* and maize, likely indicates a source of energy for meiotic processes (Dukowic-Schulze et al., 2014a). Support for this claim is provided by the fact that in *Caenorhabditis elegans* a mitochondrial protein is required for meiotic chromosome motility and correct assembly of the synaptonemal complex (Labrador et al., 2013). These data indicate that many still-unexamined cellular processes, including respiration and energy production, are needed to support the progression of meiosis, and that these processes require expression of a large number of genes.

The second hypothesis on the abundance of meiotic transcripts suggests that transcription during meiosis is promiscuous. If this were the case, it would require mechanisms allowing meiocytes to differentiate between transcripts that are needed for meiosis and those that are not. These mechanisms would be essential in order to avoid overwhelming the translation machinery and spurious synthesis of proteins whose ectopic accumulation could harm the cell. Furthermore, in addition to differentiating between essential and un-needed transcripts, post-transcriptional mechanisms could provide finer regulation of activity of transcripts required for meiosis (Caryl et al., 2000).

A hybrid model incorporating elements of the two hypotheses is also possible. Some genes, for example those essential for meiosis progression, could be tightly regulated at the RNA level. In contrast, genes whose products do not function in meiosis could be unregulated and allowed to be ectopically transcribed. However, transcripts of these genes would be prevented from being translated.

Most genome-wide studies in meiosis to-date have been focused on examining the transcriptome and very few studies addressed the proteome. Examining meiotic transcripts provides a good start for identifying genes with meiotic function. However, not all transcripts are translated into functional proteins and studying the proteome may provide a more focused view on genes acting in meiosis. Early meiotic studies in lily and tobacco, reviewed in Dukowic-Schulze and Chen (2014), used ribosome profiling to examine meiosis beyond the transcriptome level. This technique was later adapted for an extensive study of ribosome bound RNAs in yeast meiosis (Brar et al., 2012). Proteomics studies on anthers have been conducted in several plant species (Imin et al., 2001; Kerim et al., 2003; Holmes-Davis et al., 2005; Noir et al., 2005; Sanchez-Moran et al., 2005; Sheoran et al., 2006, 2007; Wang et al., 2012; Ischebeck et al., 2014), though only a few of them included anthers at meiosis (Imin et al., 2001; Kerim et al., 2003; Ischebeck et al., 2014). Studies solely focused on meiotic proteome is available in the mouse (Guo et al., 2011; Gan et al., 2013). More such studies will be needed for a complete understanding of meiosis.

## ORGANIZATION OF MEIOTIC CHROMATIN

Changes in chromatin and chromosome organization at the onset of meiosis may be responsible for the complexity of the meiotic transcriptome. Meiosis-specific histone modifications could contribute to generating high levels of active transcription (Oliver et al., 2013). In particular, the elevated expression of transposable elements observed in *Arabidopsis* and maize meiocytes could be a result of general de-repression of chromatin (Chen et al., 2010; Yang et al., 2011; Dukowic-Schulze et al., 2014a).

Chromatin undergoes drastic structural and spatial reorganization in early meiotic prophase I (Dawe et al., 1994; Zickler and Kleckner, 1998; MacQueen and Villeneuve, 2001; Kimmins and Sassone-Corsi, 2005; Yang et al., 2006; Sheehan and Pawlowski, 2009). This reorganization includes changes in histone modification patterns, chromosome condensation, and repositioning within the nucleus.

Several reports have indicated existence of meiosis-specific chromatin remodeling. This evidence includes the fact that genes involved in chromatin remodeling are expressed during anther development in rice (Deveshwar et al., 2011). Indeed, histone modification patterns have been observed to differ between mitotic and meiotic cells in *Arabidopsis,* indicating differences in the way chromatin behaves in meiosis (Oliver et al., 2013). Furthermore, histone hyperacetylation was found to be required for proper recombination and chromosome segregation in *Arabidopsis* (Perrella et al., 2010). Additional evidence for the function of chromatin remodeling in meiosis comes from the study of polyploids. In hexaploid wheat, a specific chromatin organization controlled by the *Ph1* locus aids identification of the homologs for pairing and prevents ectopic pairing and recombination between homoeologous (i.e., similar but not homologous) chromosomes (Riley, 1958; Prieto et al., 2004; Colas et al., 2008).

In addition to chromatin remodeling, structural and spatial reorganization of chromatin and chromosomes have been documented during meiotic prophase I. In maize, they have been suggested to be critical for chromosome pairing and recombination (Dawe et al., 1994). Furthermore, maize chromosomes have been observed to exhibit rapid and dynamic motility in prophase I, which is thought to facilitate their search for homologous partners (Sheehan and Pawlowski, 2009).

An impediment to elucidating the specific role of meiotic chromatin remodeling in generating the complexity of meiotic transcriptome, is the lack of understanding of how chromatin remodeling is controlled in plant meiosis at the mechanistic level. Some information in this area is, however, available in several species outside of plants. In *S. pombe*, it has been proposed that for genes with no known regulatory elements, chromatin architecture provides a system of transcriptional control (Mata et al., 2002). In *C. elegans*, mutants in *CHK-2*, a gene controlling progression of meiotic prophase and chromosome pairing, fail to spatially organize chromatin (MacQueen and Villeneuve, 2001). In mammals, there is a testis-specific mechanism that replaces the regular histone linker with a variant linker and exchanges other histones for a small arginine-rich protein called protamines (Kimmins and Sassone-Corsi, 2005). This process induces DNA compaction, affecting chromatin structure and organization and likely impacting gene expression as well. Finally, promiscuous transcription during spermatogenesis in mouse has been attributed to continuous repacking of chromatin during meiosis as well as epigenetic reprogramming that occurs in the form of waves of DNA methylation and demethylation (Soumillon et al., 2013).

The interplay known to exist between chromatin modifications and DNA methylation (Cedar and Bergman, 2009) could imply that both mechanisms contribute to changes in meiotic chromatin. Though not much is understood about DNA methylation patterns in plant meiosis, there is evidence indicating that microspores in *Arabidopsis* exhibit altered DNA methylation patterns compared to other reproductive tissues (Calarco et al., 2012). This evidence could suggest that during meiosis, there are changes in DNA methylation patterns, though more research is needed to test this hypothesis.

## REGULATION OF GENE EXPRESSION IN MEIOSIS BY SMALL RNAs

Although the nature of the transcriptional and/or post-transcriptional mechanisms hypothesized to regulate the meiotic transcriptome is unclear, it is likely that they include components of the non-coding RNA pathway (Kleene, 2001; Pang et al., 2006). Genes known to participate in the RNAi pathway have been found to be up-regulated in meiosis (Deveshwar et al., 2011). However, little is known about specific non-coding RNAs that could be involved in meiotic processes in plants, although several reports hint that such an involvement is likely. The most promising example is a novel class of secondary short interfering RNAs (siRNAs) that have been recently discovered in cereals and are preferentially expressed in the stamen (Song et al., 2011; Arikit et al., 2013). Biogenesis of these secondary siRNAs is triggered by microRNAs (miRNAs). The siRNAs are either 21 nt or 24 nt in size and produced in a phased manner, and hence named phased secondary siRNAs (phasiRNAs; Song et al., 2011). In addition to these small RNAs, several miRNAs known to be involved in transcriptional gene silencing have been detected in the meiosis RNA-seq studies of *Arabidopsis* (Mi et al., 2008; Yang et al., 2011).

Involvement of small RNAs in meiosis has also been documented outside of plants. PIWI proteins are small-RNA-binding proteins in the germline of metazoans that belong to the family of Argonaute (AGO) proteins. PiRNAs, small RNAs associated with PIWI, serve to silence transposons in the mouse male germline (Aravin et al., 2003, 2006; Girard et al., 2006; Grivna et al., 2006; Lau et al., 2006; Gu et al., 2009). Two waves of piRNAs production have been detected. The pre-meiotic wave targets transposable elements. A later wave occurs in pachytene and has a less-understood function (Li et al., 2013).

Little is known about what specifically phasiRNAs and miRNAs might do in meiosis in plants, and functional studies are needed in this area. However, there is precedence for small RNAs playing major roles in reproductive development, although not specifically during meiosis. Small RNAs have been found in both male and female gametophytes of *Arabidopsis* (Grant-Downton et al., 2009; Olmedo-Monfil et al., 2010; Borges et al., 2011) and have been implicated in germ cell specification and formation. In addition, retrotransposon-derived 21 nt-long siRNAs have been shown to regulate transposable element activity during pollen development in *Arabidopsis* (Slotkin et al., 2009).

**FIGURE 1:**
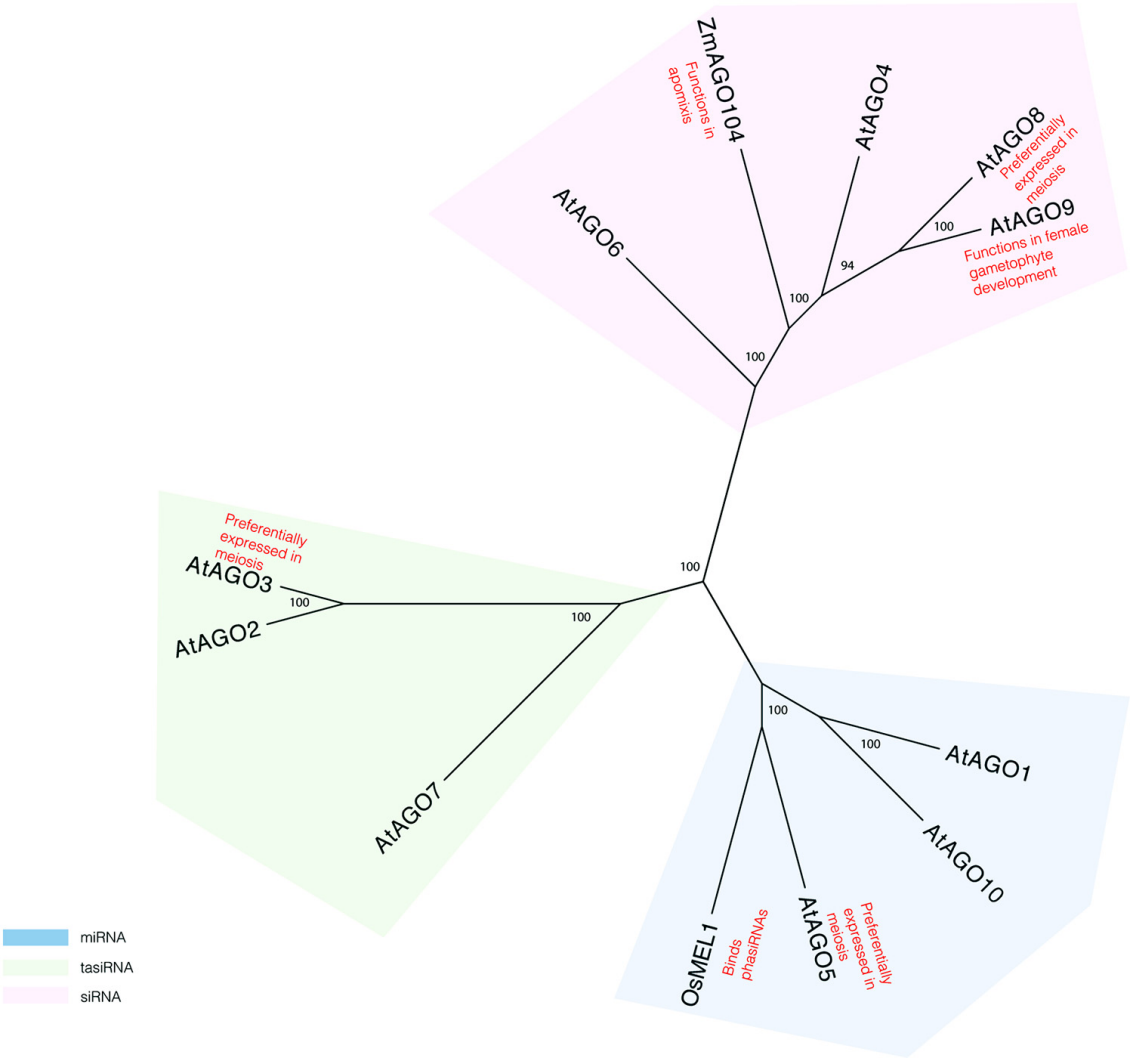
**Phylogeny reconstruction of *Arabidopsis* AGO proteins and some of their homologs in maize and rice.** Maximum likelihood bootstrap support values are displayed next to branches. Only values above 70 are shown.

More evidence for a role of the RNAi pathway in plant meiosis can be found in studies of proteins known to act in this pathway. AGO proteins, which are critical components of the pathway, have specifically been implicated in reproductive development and meiosis (**Figure 1**). AGO9 in *Arabidopsis* regulates small RNAs in the female gametophyte and acts in specifying the cell fate of the ovule (Olmedo-Monfil et al., 2010). *AGO9* mutants in *Arabidopsis* display a high frequency of chromosome entanglements during male meiosis, but do not lead to any changes in chiasmata frequency (Oliver et al., 2014). Mutations in *AGO104*, the ortholog of *AGO9* in maize, induce apomixis, a form of asexual reproduction that bypasses meiosis and fertilization (Eckardt, 2011; Singh et al., 2011). AGO104 acts to represses somatic fate of germ cells through the small RNA pathway (Eckardt, 2011; Singh et al., 2011). A rice *AGO, MEL1,* named for its arrested-at-leptotene phenotype when mutated, functions in maintaining germ cell identity and has been proposed to have a role in regulating gene expression and chromatin modifications (Nonomura et al., 2007). Recently, MEL1 was shown to bind phasiRNAs (Komiya et al., 2014), suggesting that they indeed have meiotic roles.

Transcriptome analyses of isolated *Arabidopsis* meiocytes implicate additional AGO proteins in having meiotic functions. Genes encoding AGO3 and AGO8 are preferentially expressed in meiosis, as compared to seedlings (Chen et al., 2010). However, there is no direct evidence yet that these genes affect gene silencing during meiosis. Single mutants in *AGO3* and *AGO8* do not display meiotic defects (Oliver et al., 2014). However, it is possible that analysis of double and triple mutants may provide more insight in the role of AGOs in meiosis.

## REGUATION OF GENE EXPRESSION IN MEIOSIS BY LONG NON-CODING RNAs

**FIGURE 2:**
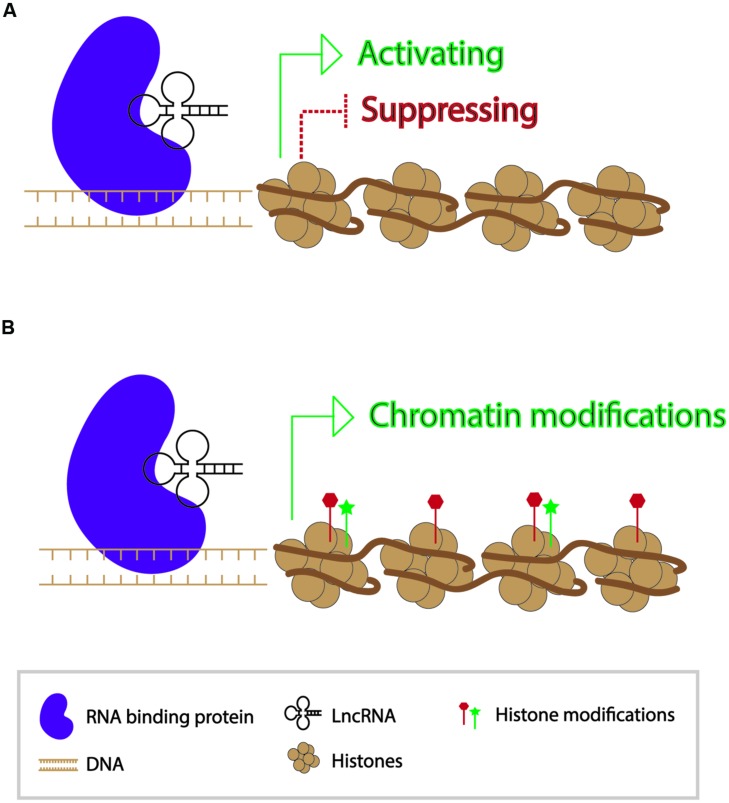
**Modes of action of long non-coding RNAs in transcriptional regulation of genes. (A)** Long non-coding RNAs can participate in gene activation and suppression. **(B)** Long non-coding RNAs can act as a guide for chromatin modifications.

In contrast to small RNAs which are 21–24 nt in length, long non-coding RNAs (lncRNAs) are generally >200 bp in length and polyadenylated (Nam and Bartel, 2012). They form secondary structures which allow them to interact with other nucleic acid molecules to function in activating and repressing genes, as well as in epigenetic modification of chromatin (Wang and Chang, 2011) (**Figure 2**). Since the discovery of the first lncRNA, Xist, in mammals and its role in X-chromosome inactivation, thousands of lncRNAs have been identified. While a number of lncRNAs have indeed been found to play roles in regulating gene expression, the functions of the vast majority of them are unknown (Lee, 2012). A transcriptome study in mouse indicated that roughly 8000 lncRNAs were expressed in spermatids and spermatocytes. Presence of these lncRNAs is not a result of ectopic read-through transcription but likely a controlled activity (Soumillon et al., 2013; Margolin et al., 2014).

Several studies have indicated that lncRNA genes are expressed during meiosis, but so far only one has been shown to have a direct meiotic role. A study by Ding et al. (2012), conducted in fission yeast, described a lncRNA that mediates pairing between homologous chromosomes during prophase I. The 1.5 kb-long lncRNA attaches to the RNA-binding domain of Mei2, a protein that is needed for entry into meiosis (Ding et al., 2012). In addition, the Mei2-bound lncRNAs accumulate at the *sme2* locus and are required for robust chromosome pairing and recombination. The authors of the study proposed a model in which pairing complexes containing lncRNAs are dispersed throughout the genome to facilitate pairing of homologous chromosomes at multiple sites. This model is intriguing because it implicates a direct structural role of lncRNAs in meiotic events, rather than just a role in regulating meiotic gene expression.

A large number of lncRNAs have been identified in genome-wide studies in *Arabidopsis* (Liu et al., 2012; Wang et al., 2014; Wang et al., submitted). However, a study by Wang et al. (submitted) represents the first time that novel lncRNAs have been identified in meiosis. The few lncRNAs that have been studied at the functional level are not meiosis-specific. Meiosis-specific lncRNAs could play roles in controlling gene expression during meiosis, either through direct activation and repression of genes or by influencing epigenetic factors acting genome-wide and/or transcriptome-wide. As many plant genomes are largely heterochromatic, these lncRNAs could also function in maintaining characteristics of heterochromatin, for example to exclude these regions from being used in the chromosome homology search, and controlling transposable elements.

## CONCLUDING REMARKS

**FIGURE 3:**
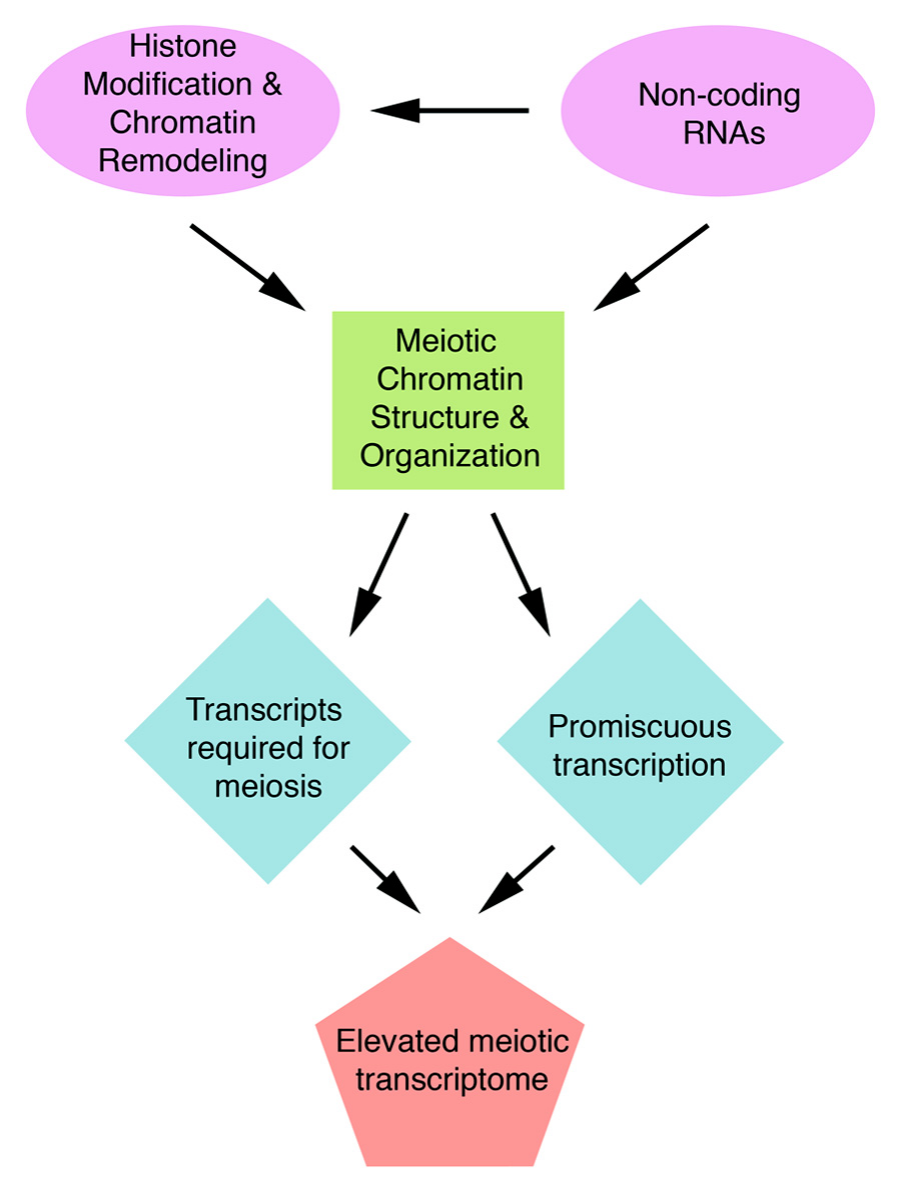
**A diagram showing potential mechanisms that could affect the meiotic transcriptome**.

Many structural proteins acting in meiosis have been examined to-date and their roles elucidated. In contrast, studies on regulation of meiotic processes are still in their infancy. One of the most important aspects of meiosis regulation is regulation of expression of meiotic genes. Recent transcriptome studies have revealed an unexpectedly high number of genes expressed during meiosis, a phenomenon that is particularly striking in plants. In *A. thaliana*, half of the annotated genes are transcribed in meiosis. This transcriptome complexity indicates that regulatory mechanism must exist to prevent chaos in gene expression. We speculate that chromatin and non-coding RNAs provide the framework to control meiotic gene expression (**Figure 3**). However, because regulation of meiotic gene expression is so poorly understood, it is possible that completely novel regulatory mechanisms could exist in meiosis. Studying the patterns and regulation of meiotic gene expression will not only provide a better understanding of meiosis but also insight into how organisms deal with the high complexity of their gene expression.

## Conflict of Interest Statement

The authors declare that the research was conducted in the absence of any commercial or financial relationships that could be construed as a potential conflict of interest.
